# Menstrual regulation: examining the incidence, methods, and sources of care of this understudied health practice in three settings using cross-sectional population-based surveys

**DOI:** 10.1186/s12905-023-02216-3

**Published:** 2023-02-17

**Authors:** Suzanne O. Bell, Mridula Shankar, Funmilola OlaOlorun, Elizabeth Omoluabi, Anoop Khanna, Danish Ahmad, Georges Guiella, Caroline Moreau

**Affiliations:** 1 Department of Population, Family and Reproductive Health, Bloomberg School of Public Health, Johns Hopkins University, 615 N. Wolfe St. Suite W4041, Baltimore, MD 21205 USA; 2 College of Medicine, University of Ibadan, Ibadan, Nigeria; 3 Center for Research, Evaluation Resources and Development, Ile-Ife, Nigeria; 4 Statistics and Population Studies Department, University of the Western Cape, Bellville, South Africa; 5 Indian Institute of Health Management Research, Jaipur, India; 6 Institut Supérieur Des Sciences de La Population (ISSP), Université of Ouagadougou, Ouagadougou, Burkina Faso; 7 Soins Et Santé Primaire, CESP Centre for Research in Epidemiology and Population Health U1018, Inserm, 94805 Villejuif, France

**Keywords:** Menstrual regulation, Abortion, Fertility, Survey methods

## Abstract

**Background:**

Menstrual regulation is a practice that may exist within the ambiguity surrounding one’s pregnancy status and has been the subject of limited research. The aim of this study is to measure the annual rate of menstrual regulation in Nigeria, Cote d’Ivoire, and Rajasthan, India, overall and by background characteristics and to describe the methods and sources women use to bring back their period.

**Methods:**

Data come from population-based surveys of women aged 15–49 in each setting. In addition to questions on women’s background characteristics, reproductive history, and contraceptive experiences, interviewers asked women whether they had ever done something to bring back their period at a time when they were worried they were pregnant, and if so, when it occurred and what methods and source they used. A total of 11,106 reproductive-aged women completed the survey in Nigeria, 2,738 in Cote d’Ivoire, and 5,832 in Rajasthan. We calculated one-year incidence of menstrual regulation overall and by women’s background characteristics separately for each context using adjusted Wald tests to assess significant. We then examined the distribution of menstrual regulation methods and sources using univariate analyses. Method categories included surgery, medication abortion pills, other pills (including unknown pills), and traditional or "other" methods. Source categories included public facilities or public mobile outreach, private or non-governmental facilities or doctors, pharmacy or chemist shops, and traditional or "other" sources.

**Results:**

Results indicate substantial levels of menstrual regulation in West Africa with a one-year incidence rate of 22.6 per 1,000 women age 15–49 in Nigeria and 20.6 per 1,000 in Cote d’Ivoire; women in Rajasthan reported only 3.3 per 1,000. Menstrual regulations primarily involved traditional or “other” methods in Nigeria (47.8%), Cote d’Ivoire (70.0%), and Rajasthan (37.6%) and traditional or “other” sources (49.4%, 77.2%, and 40.1%, respectively).

**Conclusion:**

These findings suggest menstrual regulation is not uncommon in these settings and may put women’s health at risk given the reported methods and sources used. Results have implications for abortion research and our understanding of how women manage their fertility.

## Plain English Summary

Menstrual regulation, or bringing back a late period, is an understudied practice that women may use when they are worried they are pregnant but that may be viewed as distinct from abortion. This study seeks to measure the frequency of menstrual regulation in Nigeria, Cote d’Ivoire, and Rajasthan, India, overall and by women’s characteristics and to describe the methods and sources women use. We used data from representative surveys of women aged 15–49 years old in each study setting. We asked women whether they had ever done something to bring back a late period at a time when they were worried they were pregnant, and if so, what methods and sources they used. Results indicate that menstrual regulation may be a common practice, particularly in West Africa; the observed one-year rates were 22.6 menstrual regulations per 1,000 women aged 15–49 in Nigeria and 20.6 menstrual regulations per 1000 women in Cote d’Ivoire; women in Rajasthan only reported 3.3 menstrual regulations per 1000 women per year. Menstrual regulations primarily involved traditional or “other” methods in Nigeria (47.8%), Cote d’Ivoire (70.0%), and Rajasthan (37.6%) and traditional or “other” sources (49.4%, 77.2%, and 40.1%, respectively). These findings suggest menstrual regulation is not uncommon and may put women’s health at risk given the reported methods and sources used. Results have implications for abortion research and our understanding of how women manage their fertility.

## Background

The patterns and beliefs about menstruation and its relationship to fertility, reproduction, and health are of great biological and social significance across cultures [1]. For many women,^1^ menstruation is understood as the expulsion of potentially harmful materials [2]. Qualitative research from Ghana, where various ethnic groups refer to menstruation as “cleaning the inside” or “washing the stomach”, captures this perception [3]. The women describe various practices – often involving herbs – to bring back one’s menses, the primary impetus seemingly health-related. In considering this perspective it is clear why women would seek to do something to bring back a late period. The source of amenorrhea or irregular menses may be malnourishment or other health issues, however, an alternative cause for delayed menstruation may be pregnancy. Taking actions to return a late menses when one suspects a potential pregnancy are more clearly linked to possible pregnancy and fertility regulation.

Menstrual regulation is a practice that exists within the ambiguity surrounding one’s pregnancy status and has been the subject of limited research. Most people, including many abortion researchers, view pregnancy as a simple binary, with actions taken to prevent pregnancy as contraception and those taken after a pregnancy is established as an abortion. However, this dichotomy may miss important nuances in women’s health practices, which may have fertility implications (intentional or otherwise) [4, 5].

Evidence suggests that some women view early medical terminations or actions taken to bring back a late period early in a suspected pregnancy as separate from abortion [6, 7]. Cultures, religions, and laws throughout history have frequently made distinctions between actions taken to terminate a pregnancy prior to “ensoulment” or quickening, with only those taken after this point in a pregnancy considered abortion [8–10]. This division may still be relevant for women in some settings today, where actions taken to bring back one’s period early in a suspected pregnancy are not regarded as abortion. Increasing availability of medication abortion pills may in fact have made this conceptualization more common. Research from Columbia among women who had an early medication abortion illustrates this perception: “What I did was regulate my period. I’m not going to accept that I’ve had an abortion because if I had been three or four months along, then I would have felt bad…But I don’t feel that way because I was barely a month pregnant. What I did was simply regulate my period, nothing more.’’ [11]. Other research with women in the United States depicts the view that medication abortion is more “natural”, akin to “going through [one’s] period” [12] and see menstrual regulation as an alternative to abortion that is easier on one’s emotional well-being [7].

For many, menstrual regulation brings to mind the institutionalization of this practice in Bangladesh [13]. Although abortion is illegal in the country, women have long been able to legally obtain a menstrual regulation procedure from government clinics if they have not confirmed a pregnancy and their last period was less than 10 weeks ago. Menstrual regulation in this setting benefits from the ambiguity surrounding the reason for amenorrhea in the absence of further testing. The most recent estimates indicate that menstrual regulation is common, with more than 430,000 provided in 2014 [14]. Prior evidence suggests that even more women want to obtain the procedure, with nearly a quarter of those who seek menstrual regulation being turned away for unknown reasons [15]. In this setting, women obtaining menstrual regulations are treated for amenorrhea using the same methods as those used in an early pregnancy termination. It is a common assumption that Bangladeshi women seeking menstrual regulation are all pregnant [16], however, no research has tested this. While Bangladesh is the only country where menstrual regulation is a distinct medical procedure sanctioned by the government, the practice may be more widespread. For instance, in Indonesia, although menstrual regulation is not officially an approved medical procedure and abortion remains illegal except in life endangerment of the woman, providers are more favorable towards menstrual regulation and it appears to be more common than abortion [17–19]. In this context, the distinction between menstrual regulation and abortion largely relates to timing, with early abortions considered menstrual regulations. Outside of these two countries in Asia, the authors know of no study that has sought to systematically quantify the extent and nature of menstrual regulation practices.

The current study uses population-based data from reproductive-aged women in Nigeria, Cote d’Ivoire, and the state of Rajasthan in India to address this gap in the literature. The first aim of this study is to measure the rate of menstrual regulation for the purposes of fertility regulation in these three settings and to determine the characteristics of women who report engaging in this behavior. The second aim is to describe the methods and sources of menstrual regulation for fertility management. Results will address the dearth of research related to this phenomenon and provide insight into the frequency and details of its practice.

## Methods

### Study setting

For this study, we added an abortion module to existing surveys of reproductive-aged women (15–49) in Nigeria, Cote d’Ivoire, and Rajasthan, India. We chose these settings as they were part of a multi-country study on abortion. This module included specific questions on actions taken to bring back a period when the woman was worried she was pregnant as we sought to capture the full spectrum of abortion-related actions a woman might take. These contexts represent different fertility levels and contraceptive use regimes that reflect different fertility regulating behaviors. In Nigeria and Cote d’Ivoire, the total fertility rate is high at 5.3 and 5.0 children per woman, respectively, whereas Rajasthan is approaching replacement level fertility at 2.4 [20–22]. Contraceptive use is much higher in Rajasthan (48.2%) compared to Cote d’Ivoire (25.0%) and Nigeria (24.2%), with greater reliance on female sterilization among Rajasthani women compared to women in West Africa [23–25]. Additionally, abortion is broadly legal in Rajasthan, whereas it is only legal to save a woman’s life or in the case of rape in Cote d’Ivoire, and in most of Nigeria, only legal to save a woman’s life. Evidence from Nigeria and Cote d’Ivoire suggests an annual induced abortion rate of 46 and 41 per 1000 women of reproductive age in 2017, respectively, whereas the abortion rate in Rajasthan is estimated at 23 per 1000 in 2017 [26–28]. Medication abortion pills are also more widely available in India than West Africa. As such, the study contexts provide two distinct settings in which to examine the potential role of menstrual regulation in women’s fertility regulation.

### Data

Data come from an existing multi-country project called Performance Monitoring for Action (PMA). PMA conducts annual population-based family planning surveys in several African and Asian countries. PMA uses a multi-stage stratified cluster sampling design with probability proportional to size selection of clusters, which are comprised of approximately 200 households each. Interviewers then map and list all households within selected clusters and randomly sample 35 households per cluster (40 in Lagos, Nigeria). The surveys are conducted regularly in order to track key family planning indicators and sample size determination is based on the sample required to estimate the modern contraceptive rate with a 3 percentage point precision. The sampling design is described in more detail elsewhere [29].

In-country partners conducted data collection from April through May 2018 in Nigeria, July through August 2018 in Cote d’Ivoire, and April through June 2018 in Rajasthan. Interviewers residing within or nearby selected clusters conducted surveys in English, Hausa, Igbo, Yoruba, or Pidgin in Nigeria, French in Cote d’Ivoire, and Hindi in Rajasthan. Local dialects were used to improve comprehension when necessary. All females aged 15–49 who were usual residents of or slept the prior night in a selected household were eligible to participate after providing informed consent. In accordance with local ethical approvals, respondents provided verbal informed consent in Nigeria and Cote d’Ivoire and written informed consent in Rajasthan. The Johns Hopkins University Bloomberg School of Public Health provided ethical approval for this study (8308), as did the National Health Research Ethics Committee of Nigeria (NHREC/01/01/2007–02/01/2018C), the Comite National D’ Ethique de la Recherche (CNER) in Cote d’Ivoire (N/Ref: 036–18/MSHP/CNER-kp), and the Indian Institute of Health Management Research (IIHMR) Institutional Review Board for Protection of Human Subjects in Rajasthan (Feb 2018 1).

In the abortion module, interviewers asked women whether they had ever done something to bring back their period at a time when they were worried they were pregnant (i.e. menstrual regulation for the purpose of fertility regulation, which we are simply referring to as menstrual regulation). Researchers’ understanding of this practice and the specific language that captured it emerged during discussions with female data collectors from study areas in Nigeria during the pilot training. The women made a distinction between actions women take when their period is late and they suspect they may be pregnant but have not confirmed it and when a pregnancy is more established or confirmed through a pregnancy test or other pregnancy symptoms. We conducted similar discussions with interviewers and respondents involved in the pilot training in the other study countries and affirmed the importance of asking questions about menstrual regulation in the other contexts as well. We confirmed comprehension and interpretation of this language and the corresponding translations during piloting in each country. The prelude to the abortion module, which included the menstrual regulation questions, framed the content with regards to actions women take when they become pregnant at a time when they cannot or do not want to be pregnant in order to minimize reporting of miscarriage. Interviewers asked about the year of the menstrual regulation, whether the woman did multiple things in the process of regulating her menses, and the method(s) and source(s) used.

### Analysis

To achieve aim one, we estimated the overall one-year incidence of menstrual regulation. Due to survey constraints, we were unable to collect data on event month, only year. Thus, to calculate the one-year incidence we included reported events that occurred between January 1, 2017 and the date of the survey in 2018 and divided by the number of woman-years between January 1, 2017 and the date of the survey in 2018; each respondent contributed on average 1.28 woman-years in Nigeria, 1.55 woman-years in Cote d’Ivoire, and 1.35 woman-years in Rajasthan. We then multiplied the value by 1000 to convert the estimate into a one-year incidence per 1000 women age 15 to 49; we scaled the standard errors using the same approach. We calculated the menstrual regulation one-year incidence rates overall and by background characteristics for each setting, including categorical variables for age (5-year age groups), education (never, primary, secondary, higher), marital status (currently married/cohabiting, divorced or separated/widowed, never married) wealth quintile, parity (0, 1–2, 3–4, 5 +), residence (urban, rural), as well as country specific measures for religion, ethnicity (caste in Rajasthan), and in Nigeria, state. To evaluate whether menstrual regulation rates different by respondent characteristics we used adjusted Wald tests. There were little to no missing data on study variables in each setting (1% or less), thus we simply omitted these observations in corresponding calculations. However, for the menstrual regulation source variable we coded methods other than surgery and pills (i.e., all traditional methods, home remedies, or other methods) as being accessed from traditional or other non-clinical sources given we did not ask about their source directly.

To achieve aim two, we examined the distribution of menstrual regulation methods and sources using univariate analyses. Method categories included surgery (we did not probe to determine the specific abortion surgery given the limitations of respondent knowledge regarding the procedure), medication abortion pills (misoprostol with or without mifepristone), other pills (antibiotics, antimalarial medicines, and other pills) as well as those where the pill type was unknown, and traditional or "other" methods (herbs, homemade remedies, injections, alcohol, etc.). Source categories included public facilities or public mobile outreach, private or non-governmental facilities or doctors, pharmacy or chemist shops, and traditional or "other" sources (markets, shops, friends or relatives). We did not ask women where they obtained their method if it was not a surgery or any type of pill, thus for traditional and other methods we assumed the source was not a public facility, private facility or doctor, or pharmacy or chemist and categorized the source as “other”.

We conducted all analyses in Stata version 15.1 using survey design weights to account for the complex sampling strategy and household and female non-response and calculated robust standard errors to adjust for clustering.

## Results

A total of 11,106 women of reproductive age completed the survey in Nigeria, 2738 women in Cote d’Ivoire, and 5832 women in Rajasthan, with a response rate of approximately 98% in each setting. Respondent characteristics are provided elsewhere [30].

Results indicate varying levels of menstrual regulation with an overall one-year incidence of 22.6 per 1000 women aged 15–49 in Nigeria, 20.6 in Cote d’Ivoire, and 3.3 in Rajasthan (Tables 1, 2, 3). Women aged 20 to 29 in all three countries had the highest rates of menstrual regulation, and women with more education generally had higher incidences, with those who attended secondary education or higher having the highest rates (Tables 1, 2, 3). In Nigeria and Cote d’Ivoire, women who had never been married had the highest rates of menstrual regulation at 33.9 and 25.6 per 1000, respectively (although the difference by marital status was not statistically significant in Cote d’Ivoire). In Rajasthan, the menstrual regulation rate among unmarried women was similar but slightly lower than the rate among currently married/cohabiting women (2.8 and 3.5, respectively). Menstrual regulation incidence was significantly higher among women residing in urban areas in Nigeria, while the difference by residence was not significant in Rajasthan or Cote d’Ivoire. There was no consistent pattern across settings with regard to wealth and parity, however, greater parity was significantly associated with lower rates of menstrual regulation in Nigeria. Additionally, there were statistically significant relationships with regard to ethnicity, religion, and state in Nigeria.

**Table 1:** One-year incidence of menstrual regulation (per 1000) overall and by characteristics of female respondents aged 15–49, Nigeria (N = 11,106)*

	Rate	95% CI		P-value
Age				
15–19	14.1	7.2	21.0	0.001
20–24	42.4	25.0	59.8	
25–29	30.6	16.8	44.4	
30–34	20.3	11.6	29.0	
35–39	19.3	11.8	26.8	
40–44	7.2	1.6	12.8	
45–49	12.6	4.1	21.1	
Education				
Never	8.6	2.9	14.3	0.004
Primary	20.6	12.0	29.2	
Secondary	26.0	16.4	35.7	
Higher	28.3	17.3	39.3	
Marital status				
Currently married/cohabiting	17.5	12.0	23.1	0.002
Divorced or separated/widowed	16.8	6.6	26.9	
Never married	33.9	21.6	46.1	
Religion of household				
Catholic	24.2	13.3	35.1	0.001
Other Christian	33.8	20.6	46.9	
Muslim	9.9	6.3	13.5	
Other	15.6	0.0	33.9	
Ethnicity				
Hausa	7.3	3.1	11.6	< 0.001
Igbo	28.2	18.7	37.6	
Yoruba	21.0	11.8	30.2	
Other	27.6	14.2	41.1	
Wealth				
Poorest	13.2	1.8	24.6	0.474
Second poorest	24.6	13.2	36.0	
Middle	30.9	15.0	46.7	
Second wealthiest	24.3	14.5	34.2	
Wealthiest	22.7	12.8	32.5	
Parity				
0	29.1	18.5	39.7	0.025
1–2	25.8	15.1	36.5	
3–4	17.5	10.8	24.2	
5 +	12.1	6.9	17.3	
Residence				
Rural	13.2	6.5	19.9	0.010
Urban	29.7	19.1	40.4	
State				
Anambra	31.2	16.9	45.6	< 0.001
Kaduna	12.7	6.1	19.4	
Kano	2.3	0.0	4.7	
Lagos	23.4	15.6	31.2	
Nasarawa	12.9	4.4	21.4	
Rivers	39.0	22.2	55.8	
Taraba	30.2	0.0	75.5	
Overall	22.6	15.7	29.5	–

*Estimates weighted; p-value from Adjusted Wald test

**Table 2:** One-year incidence of menstrual regulation (per 1000) overall and by characteristics of female respondents aged 15–49, Cote d'Ivoire (N = 2738)*

	Rate	95% CI		P-value
Age				
15–19	16.5	7.3	25.8	0.515
20–24	27.0	15.7	38.4	
25–29	22.3	6.3	38.3	
30–34	17.0	0.5	33.4	
35–39	20.9	4.9	36.9	
40–44	22.4	3.0	41.8	
45–49	15.2	0.0	33.2	
Education				
Never	14.5	6.3	22.7	0.201
Primary	20.8	11.1	30.4	
Secondary	29.0	15.0	43.1	
Higher	33.2	3.8	62.7	
Marital status				
Currently married/cohabiting	18.1	10.1	26.0	0.339
Divorced or separated/widowed	22.4	0.0	45.1	
Never married	25.6	15.0	36.2	
Religion of household				
Muslim	10.1	3.3	16.8	0.079
Catholic	33.7	16.8	50.6	
Evangelical	19.8	4.1	35.5	
Other	32.0	9.7	54.4	
No religion	21.0	8.7	33.2	
Ethnicity				
Akan	30.0	15.1	45.0	0.153
Mande (North and South)	15.7	3.7	27.8	
Gur	24.3	8.1	40.6	
Other Ivoirian	17.9	1.9	34.0	
Other non-Ivoirian	8.4	0.1	16.8	
Wealth				
Poorest	18.7	1.4	36.1	0.982
Second poorest	17.8	4.9	30.7	
Middle	23.7	10.5	36.9	
Second wealthiest	20.5	4.3	36.8	
Wealthiest	22.3	9.8	34.7	
Parity				
0	19.3	7.0	31.5	0.479
1–2	26.3	14.8	37.8	
3–4	16.8	6.3	27.2	
5 +	17.3	1.7	32.9	
Residence				
Rural	22.7	8.2	37.3	0.677
Urban	19.2	11.1	27.4	
Overall	20.6	13.1	28.1	–

*Estimates weighted; p-value from Adjusted Wald test

**Table 3:** One-year incidence of menstrual regulation (per 1000) overall and by characteristics of female respondents aged 15–49, Rajasthan, India (N = 5832)*

	Rate	95% CI		P-value
Age				
15–19	2.4	0.0	6.4	0.014
20–24	7.9	2.7	13.0	
25–29	3.4	0.9	5.9	
30–34	2.8	0.0	6.9	
35–39	1.0	0.0	3.3	
40–44	1.3	0.0	3.2	
45–49	0.3	0.3	0.4	
Education				
Never	1.5	0.2	2.8	0.168
Primary	2.9	0.3	5.4	
Secondary	7.4	1.2	13.5	
Higher	4.4	0.0	9.7	
Marital status				
Currently married/cohabiting	3.5	1.7	5.2	0.000
Divorced or separated/widowed	0.0	–	–	
Never married	2.8	0.0	6.6	
Religion of household				
Hindu	2.8	1.3	4.3	0.530
Muslim	5.8	0.0	11.8	
Other	8.6	0.0	26.7	
Caste of household				
Scheduled caste	3.4	0.0	7.0	0.102
Scheduled tribe	5.7	0.0	11.5	
Other backward caste	3.4	1.0	5.8	
General	1.3	0.6	1.9	
Wealth				
Poorest	4.3	0.0	8.6	0.566
Second poorest	1.5	0.0	3.3	
Middle	2.5	0.0	5.1	
Second wealthiest	3.5	0.0	7.3	
Wealthiest	4.2	0.6	7.9	
Parity				
0	3.3	0.4	6.2	0.001
1–2	3.5	1.2	5.8	
3–4	3.8	0.8	6.8	
5 +	0.0	0.0	0.0	
Residence				
Rural	2.9	1.2	4.5	0.538
Urban	4.0	0.9	7.0	
Overall	3.3	1.7	4.8	–

*Estimates weighted; p-value from Adjusted Wald test

The methods and sources women predominantly used to regulate their period in each of the settings were similar (Table 4). The most common menstrual regulation methods women used were traditional or “other” methods at 47.8% in Nigeria (primarily traditional methods like herbs, followed by alcohol and injections), 70.0% in Cote d’Ivoire (primarily herbs), and 37.6% in Rajasthan (primarily home remedies). The next most common method was pills other than medication abortion pills, which included pills of unknown type (43.9%, 21.5%, and 26.0%, respectively). In Rajasthan, a significant proportion of menstrual regulations involved misoprostol with or without mifepristone (24.9%) compared to Nigeria (4.8%) and Cote d’Ivoire (2.3%). Women in Rajasthan were also more likely to report using surgery to bring back their period (11.5%) compared to Nigeria (3.5%) and Cote d’Ivoire (6.2%).

**Table 4:** Menstrual regulation method among respondents aged 15–49, by country

	Nigeria		Cote d'Ivoire		Rajasthan	
	%	N	%	N	%	N
Surgery	3.5	19	6.2	10	11.5	10
Mifepristone/misoprostol pills	4.8	30	2.3	4	24.9	22
Non-medication abortion pills/pill type unknown	43.9	258	21.5	45	26.0	23
Traditional/other methods	47.8	266	70.0	137	37.6	32
Total	100.0	573	100.0	196	100.0	87

Similar to methods, the most common sources of menstrual regulation were consistent across study contexts. Since women most often used traditional or "other" methods to regulate their periods in all three settings, traditional or "other" sources were the most frequently used source at 49.4% in Nigeria, 77.2% in Cote d’Ivoire, and 40.1% in Rajasthan (Table 5). The next most common source for menstrual regulation methods was pharmacies or chemists in Nigeria (36.9%), Cote d’Ivoire (12.5%), and Rajasthan (28.8%). In Rajasthan, a substantial proportion of menstrual regulations relied on private or non-governmental facilities or doctors (24.4%), which was in contrast to the relative infrequency of this source in Nigeria (7.8%) and Cote d’Ivoire (3.1%).

**Table 5:** Menstrual regulation source among respondents aged 15-49, by country

	Nigeria		Cote d'Ivoire		Rajasthan	
	%	N	%	N	%	N
Public facility or mobile outreach	5.9	49	7.2	13	6.7	8
Private/non-governmental organization facility or doctor	7.8	43	3.1	5	24.4	17
Pharmacy/chemist	36.9	200	12.5	23	28.8	28
Traditional/other source	49.4	277	77.2	157	40.1	34
Total	100.0	569	100.0	198	100.0	87

## Discussion

Findings suggest menstrual regulation is common in West Africa while perhaps less so in India. One-year menstrual regulation rates ranged from 3.3 per 1000 reproductive-aged women in Rajasthan to 20.6 in Cote d’Ivoire and 22.6 in Nigeria. Women most often used traditional or “other” methods to regulate their period, followed by pills. To access these methods women predominantly relied on traditional or other non-clinical sources; pharmacies and chemists were the second most common source. Rajasthan was distinct in that use of medication abortion pills and private or non-governmental organization providers comprised a substantial portion of the menstrual regulation sources whereas these sources were much less common in the West African countries. The lower rate of menstrual regulation in Rajasthan may reflect the legality and increased availability of abortion services (particularly medication abortion pills at pharmacies) [31, 32], which women may be more likely to report simply as abortions. Rajasthan is also a context where contraceptive use is higher and fertility is lower than the West African study countries, thus the lower rate of menstrual regulation for the purpose of fertility regulation may reflect Rajasthan being further along in the fertility transition than Nigeria and Cote d’Ivoire; abortion rates (and presumably rates of menstrual regulation for fertility regulating purposes) tend to be lower in settings the have completed the fertility transition [33].

Previous literature on menstrual regulation has focused primarily on Bangladesh, where it constitutes a legal medical procedure. The most recent facility-based estimates suggest a rate of 10 menstrual regulations per 1,000 women aged 15-49 in 2014, however, many women may go on to obtain these menstrual regulations from informal providers, self-manage their menstrual regulation, or seek a clandestine abortion [14, 15]. Research from Ghana suggests a similar level of menstrual regulation at 10 per 1000 reproductive-aged women per year [34]. Our findings suggest that women’s reliance on menstrual regulation to manage their fertility in the context of suspected pregnancy is relevant in multiple geographies other than Bangladesh and Indonesia, indicating this practice may be much more widespread than previously assumed.

The potential risk of maternal morbidity and mortality associated with these menstrual regulation practices are largely unknown. Our data suggest this behavior is mostly conducted in the informal health sector and involves non-recommended pills (i.e. not medication abortion pills). The safety profile depends on the actual underlying reason for delayed menses and the gestational age in the case of pregnancy. With increasing diffusion of medication abortion pills, we may see a shift towards safer practices when women use these pills to regulate their fertility. We saw this in the Rajasthan data, where medication abortion pills are more widely available as a result of the legality of abortion. However, we may anticipate greater availability and awareness of misoprostol (and mifepristone) in coming years regardless of legality in other settings as well.

These findings have implications for abortion research. Since we were unable to determine whether the women in this study were in fact pregnant at the time they did something to bring back their period, it is not obvious how best to incorporate the menstrual regulation data into estimates of induced abortion incidence. Including all period regulations would likely produce an overestimate, particularly in Nigeria and Cote d’Ivoire where availability and use of effective medication abortion pills is less common. However, excluding all of them would likely fail to account for many instances where women have undergone events that would be considered by researchers as induced abortions. In prior work, we incorporated half of the period regulations to the pregnancy removal data to produce a final estimate of the one-year induced abortion incidence in Nigeria and Cote d’Ivoire [27, 28]. In more recent work we asked additional follow-up questions to explicitly ascertain the motivation for the menstrual regulation, likelihood of pregnancy, and whether the woman successfully brough back her period [35]. Future work that seeks to produce estimates of induced abortion incidence would benefit from collecting period regulation data, including additional details on the duration of menstrual delay, how certain respondents are in their pregnancy suspicions, and/or whether they have taken a confirmatory pregnancy test. More broadly, further research is needed to determine how this practice fits into other pre- and post-coital fertility regulating behaviors that women might use to manage their fertility.

An alternative explanation of these findings that again has implications for abortion research is that the menstrual regulation questions offer a less threatening way to refer to or conceptualize one’s abortion experience. Especially in settings like Nigeria and Cote d’Ivoire, where abortion is highly legally restricted and stigmatized, the ambiguity surrounding these practices and whether the woman was pregnant at the time can be productive, shielding women from social, psychological, and legal repercussions if she were known to have had an abortion [4]. As such, women may be motivated to reframe their experience in a manner that is more acceptable to themselves and others. This may be particularly true in a survey context, where respondents may feel greater social desirability pressure to not admit to engaging in stigmatized behaviors like abortion [36]. Menstrual regulation may more accurately reflect some respondents’ view of pregnancy as a continuous rather than binary state, similar to other phenomenon, like marriage, which Africans may view as a process, not a distinct event [37]. Future research should explore the extent to which these ideas might partially explain our findings.

This study has a number of strengths, the main one being that it is the first study to systematically evaluate the incidence, methods, and sources of menstrual regulation outside of Bangladesh where it is uniquely codified as a medical procedure for treatment of irregular menses or amenorrhea. The data we used are representative of reproductive-aged women in each setting, providing population-based estimates of the extent of this behavior and the characteristics of the women practicing it. Additionally, the contemporaneous collection of these data in three different settings using similar instruments and training methodologies enabled cross-country comparisons of this practice across geographically and culturally diverse regions with different abortion legality and availability of medication abortion pills.

Despite these strengths, our study is not without limitations. The main limitation is that we are unable to validate the incidence estimates. Similar to abortion, this practice may also be viewed as stigmatizing to some women who think it is no different from abortion, which would mean the observed rates are an underestimate. As part of the broader study, we also collected information on respondents’ closest female friends and their experience with period regulation (as well as pregnancy removal). Friend estimates of menstrual regulation incidence were similar to that of the respondents in Nigeria (28.3 per 1000) and Cote d’Ivoire (20.8) whereas the friend estimate was nearly four times greater than the respondent estimate in Rajasthan (11.8) [27, 28, 30]. In the case of Rajasthan, this also led to a relatively small number of self-reported menstrual regulations on which we could examine the method and source used. In addition to potential underreporting as a result of social desirability bias, the extent of menstrual regulation may be even greater given we framed these questions in terms of a time when the woman was worried she might be pregnant. To the extent that women use menstrual regulation for broader health reasons, as some other evidence suggests [3, 4], our data would not capture these circumstances of menstrual regulation. Another concern is the potential for misclassification in the methods and sources of the period regulations or misinterpretation of the questions. Many women were not able to provide details about the specific pills they took, which would tend to misclassify medication abortion pill use as other pills. Additionally, while we checked for correct interpretation of the period regulation translated phrases during the pilot studies in each country, the novel framing of these questions could have unintentionally captured some miscarriages or emergency contraception use. Lastly, since abortion was the focus of the broader study and we were primarily interested in the most recent event that could potentially be interpreted as such, for women who reported a pregnancy removal and a period regulation, we only collected method and source details on the most recent of the two events. As such, we were unable to report on the details of 94 period regulations in Nigeria, 20 in Cote d’Ivoire, and 21 in Rajasthan as these women all had reported a more recent pregnancy removal.

## Conclusion

These menstrual regulation findings suggest we have much more to learn with regard to this understudied health behavior. It may be the case that menstrual regulation better captures early abortions and medication abortions if women view them differently from later abortions or abortions using other methods (e.g., surgery) [5]. However, the motivation behind and interpretation of menstrual regulation is culturally and individually determined, thus the practice may not represent the same thing across populations or over time [4, 13]. It is apparent from our results that this practice needs further study to better elucidate the extent to which and for whom menstrual regulation represents a practice distinct from contraceptive method use (e.g., emergency contraception) or abortion versus a less stigmatizing way to conceptualize an abortion. This research has implications for not only abortion research but for our understanding of women’s health practices and the ways in which they regulate their fertility. This work may also have clinical implications to the extent that women are seeking menstrual regulation services or treatment for complications from menstrual regulation in facilities.

## Data Availability

Data underlying this study include PMA household/female data for Nigeria Round 5 (https://doi.org/10.34976/b5eg-a867), Cote d’Ivoire Round 2 (https://doi.org/10.34976/716t-1697), and Rajasthan Round 4 (https://doi.org/10.34976/87gc-wn69), which are available at www.pmadata.org/data/request-access-datasets.

## Footnotes

1 We used the term “women” throughout the paper because that is the language used in our sources and in our data, where respondents had to self-identify as a woman to participate in the survey. However, we recognize that people other than those who identify as women can menstruate or become pregnant.
